# Effect of Statin Intensity on Cardiovascular Outcomes and Survival Following Coronary Artery Bypass Grafting

**DOI:** 10.1002/clc.70170

**Published:** 2025-07-01

**Authors:** Iftikhar Ali Ch, Khurram Nasir, Uzair Majeed, Azhar Chaudhry, Muhammad Abdullah, Ali Haider, Asadullah Jamal, Anum Hussain, Hammad Iftikhar, Salman Khalid, Pei‐Tzu Wu, Yusuf Shah, Arham Niaz, Muhammad Siddique, Naeem Tahirkheli

**Affiliations:** ^1^ South Oklahoma Heart Research Oklahoma City OK USA; ^2^ SSM Health Saint Anthony Hospital Oklahoma City OK USA; ^3^ University of Oklahoma Oklahoma City Oklahoma; ^4^ Division of Cardiovascular Prevention and Wellness, Department of Cardiology Houston Methodist DeBakey Heart and Vascular Center Houston TX USA; ^5^ Armed Forces Institute of Cardiology Rawalpindi Pakistan; ^6^ Doctor of Physical Therapy Program Southern California University of Health Sciences Whittier California

**Keywords:** coronary artery bypass graft, high intensity statin, mortality, statin

## Abstract

**Background:**

High‐intensity statins are recommended for patients with chronic coronary artery disease, with reports suggesting improved clinical outcomes. However, recent findings in coronary artery bypass graft (CABG) patients question whether a treat‐to‐target low density lipoprotein (LDL) approach is non‐inferior to high‐intensity statin therapy.

**Methods:**

This single‐center observational study analyzed all CABG only (*n* = 1854) procedures performed between 2013 and 2015. Patients were divided into three groups based on statin prescription: high‐intensity statin therapy (atorvastatin ≥ 40 mg or rosuvastatin ≥ 20 mg), low/moderate‐intensity statin therapy, and a no‐statin group. The primary outcome measured was major adverse cardiovascular events (MACE), a composite of post‐CABG acute coronary syndrome, cerebrovascular accident and cardiovascular mortality.

**Results:**

No‐Statin group had significantly higher incidence of MACE compared to statin group (14.2% vs 8.9%; odds ratio (OR) 1.60, 95% confidence interval (CI) 1.055–2.427, *p* = 0.029). Low/moderate‐intensity therapy (*n* = 1301) was associated with a numerically higher overall rate of MACE compared to high‐intensity therapy (*n* = 397) but was not statistically significant (9.6% vs 6.6%; OR 1.45, CI 0.961–2.172, *p* = 0.073). Beyond 2 years post‐CABG, low/moderate intensity statin use was associated with a significant higher incidence of MACE (9.1% vs 5.3%; OR 1.72, 95% CI 0.993–2.978, *p* = 0.047) compared to high intensity statins.

Patients who received high‐intensity statin therapy had the lowest LDL levels (82.21 ± 41.85 mg/dL), compared to those on low/moderate‐intensity statins (90.84 ± 45.89 mg/dL) and no‐statin group (104.83 ± 38.93 mg/dL, *p* < 0.001).

**Conclusion:**

High‐intensity statin therapy following CABG is associated with improved long‐term clinical outcomes compared to low‐ or moderate‐intensity statin regimens.

## Summary

- **What's New?**
- While statin use overall was associated with a significant reduction in major adverse cardiovascular events compared to no statin therapy, high‐intensity statins demonstrated a trend toward superior outcomes compared to low/moderate‐intensity statins, achieving statistical significance in the later follow‐up period.
- The study provides real‐world evidence from a large, single‐center cohort, supporting the sustained benefit of intensive lipid‐lowering therapy in post‐CABG patients.
- **What Are the Clinical Implications?**
- Statin therapy should be strongly considered in all patients undergoing CABG to reduce the risk of long‐term cardiovascular events.
- Among statin users, high‐intensity statins may offer additional long‐term benefit, particularly after the second year following surgery, and should be prioritized when clinically tolerated.
- Patients who are unable to tolerate statin therapy may benefit from alternative lipid‐lowering agents, as this study demonstrated that individuals not receiving statins experienced worse clinical outcomes and had significantly higher levels of total cholesterol and LDL.

## 1 Introduction

### 1.1 Background

High‐intensity statins are widely recommended for patients with chronic coronary artery disease (CAD) due to their well‐documented role in reducing cardiovascular morbidity and mortality [1] However, in the context of coronary artery bypass grafting (CABG), the extent to which statin intensity influences short and long‐term outcomes remains a subject of ongoing debate. While lipid‐lowering therapy is central to secondary prevention [2], recent discussions have raised questions about whether high‐intensity statins confer additional benefits over moderate‐intensity therapy beyond Low density lipoprotein (LDL) reduction alone in patients undergoing CABG. Despite existing guidelines advocating for aggressive lipid control in CABG patients, the clinical impact of different statin intensities on postsurgical cardiovascular outcomes remains uncertain [3].

### 1.2 Current Knowledge

Dyslipidemia plays a central role in the development of atherosclerosis and remains a major therapeutic target in cardiovascular disease management. High‐intensity statin therapy has been shown in multiple large‐scale randomized controlled trials (RCT) and meta‐analyses to effectively reduce LDL by ≥ 50%, significantly lowering the risk of major adverse cardiovascular events (MACE) in patients with chronic coronary disease (CCD). For individuals unable to tolerate high‐intensity statins, moderate‐intensity therapy is recommended to achieve a 30%–49% reduction in LDL, also contributing to MACE risk reduction [4, 5].

Despite robust evidence supporting statin use in CCD, data specific to post‐ CABG patients remain limited. The 2018 ACC/AHA cholesterol guidelines advocate for high‐intensity statin therapy in nearly all individuals with clinical atherosclerotic cardiovascular disease, including those post‐CABG. An exception is made for patients over 75 years old, due to the underrepresentation of this group in high‐intensity statin trials and concerns over polypharmacy and tolerability [2]. Nonetheless, clinical experience and evidence evaluating the impact of high‐intensity statin use initiated early after CABG remain sparse.

A meta‐analysis including CABG patients reported that more intensive statin regimens produced a 0.51 mmol/L greater reduction in LDL‐C at 1 year, resulting in a 15% reduction in major vascular events and a 13% decline in coronary death or nonfatal myocardial infarction [5]. The Post‐CABG trial further showed that aggressive LDL‐C lowering (< 100 mg/dL) significantly slowed vein graft disease progression [6]. Long‐term follow‐up from this trial demonstrated that intensive lipid‐lowering led to a 30% reduction in repeat revascularization and a 24% decrease in composite clinical outcomes over 7.5 years compared to moderate therapy [7]. Moreover, post hoc analyses from randomized trials comparing statin intensity in CCD patients have observed higher MACE rates in CABG recipients compared to those treated with percutaneous coronary intervention over 2 years. Although intensive statin therapy reduced the risk of cardiovascular events in both subgroups, the effect did not reach statistical significance in CABG patients (HR 0.84, *p* = 0.27), likely due to smaller sample sizes, while the benefit was significant in non‐CABG patients (HR 0.86, *p* = 0.016) [8].

### 1.3 Gaps in Knowledge

While these studies provide favorable evidence supporting the use of high‐intensity statins, particularly in CCD populations, comprehensive and dedicated evidence remains sparse in patients undergoing CABG. Many of the existing RCTs and meta‐analyses included CABG patients as part of broader cardiovascular cohorts, often without predefined subgroup analyses or adequate representation of early postoperative outcomes. Literature review that patients recruited were well beyond their surgical dates and may not reflect true impact of statin therapy in these patients [4, 5, 8]. To date, no randomized controlled trial has conclusively shown that high‐intensity statins provide superior outcomes compared to low/moderate‐intensity statins in CABG patients. As a result, the role of statin intensity specifically in the immediate and long‐term post‐CABG period remains incompletely understood, underscoring the need for more targeted investigation in this high‐risk population.

### 1.4 Hypothesis and Aims of Study

We aimed to evaluate the impact of statin intensity on both lipid profile optimizations —specifically the achievement of target LDL levels—as well as short and long‐term clinical outcomes following CABG. We hypothesized that high‐intensity statin therapy would be associated with superior reductions in LDL and confer greater clinical benefit, including lower rates of MACE and perhaps improved survival, compared to low‐ or moderate‐intensity statin regimens.

## 2 Methods

This single‐center, retrospective observational study analyzed all CABG procedures performed between 2013 and 2015. This is a sub‐analysis of a previously published observation study evaluating the role of antiplatelet therapy after CABG. The original study design excluded patients undergoing concomitant valve surgery and those requiring long‐term anticoagulation after CABG. For this analysis, patients' post‐CABG medication lists were reviewed to determine the type and dose of statin therapy. Patients were divided into three groups based on their post‐surgery statin prescription: high‐intensity statin therapy (atorvastatin ≥ 40 mg or rosuvastatin ≥ 20 mg), low/moderate‐intensity statin therapy, and a no‐statin group. Additionally, lipid profiles for each patient were entered into database for evaluation between one to 3 months after surgery.

## 3 Primary Outcome

The primary outcome was the difference in the incidence of major adverse cardiovascular events (MACE) among the study groups. MACE was defined as a composite of cardiovascular mortality, post‐CABG acute coronary syndrome (ACS) and cerebrovascular accident (CVA).

## 4 Secondary Outcomes

Secondary outcomes included cardiovascular mortality, all‐cause mortality, post‐CABG acute coronary syndrome (ACS), cerebrovascular accident (CVA), revascularization, and between‐group differences in lipid parameters including LDL, total cholesterol, and high‐density lipoprotein (HDL) levels.

## 5 Selection Criteria

Inclusion:

1. All CABG only surgeries performed at Oklahoma heart hospital between 2013 and 2015.

Exclusion:

1. Noncompliance with follow‐up at our center.
2. Patients who moved out of state after surgery.
3. Early postoperative death – within 7 days of CABG surgery.

## 6 Statistical Analysis

Descriptive statistics were used to summarize patient characteristics across the three statin therapy groups. Continuous variables were reported as mean ± standard deviation and compared using one‐way analysis of variance (ANOVA). Categorical variables were presented as frequencies and percentages and compared using the Chi‐square test.

To evaluate the association between post‐CABG statin intensity and clinical outcomes, time‐to‐event outcomes, such as mortality or major adverse cardiac events, were analyzed using Kaplan‐Meier survival curves with the log‐rank test as well as with the pairwise comparison. Multivariable logistic regression was performed to calculate adjusted odds ratios (OR) with 95% CIs. Statistical significance was set at *p* < 0.05. Statistical analyses were performed using SPSS v29.0.

## 7 Results

### 7.1 Patient Characteristics

The study included 1877 post‐CABG patients, with 165 (8.7%) not receiving statins, 1,311 (69.9%) on low/moderate‐intensity statins, and 401 (21.3%) on high‐intensity statins. The mean age was significantly different across groups (*p* = 0.006), with patients in the no‐statin group being the oldest (67.04 ± 10.84 years) compared to those on low/moderate‐intensity (65.23 ± 9.90 years) and high‐intensity statins (64.14 ± 9.49 years). Males comprised the majority of the study population, with the highest proportion in the high‐intensity statin group (76.0%), followed by low/moderate‐intensity (74.1%), and the no‐statin group (62.4%) (*p* = 0.003). Patients on high‐intensity statins had the highest BMI (31.57 ± 5.79 kg/m²) and had higher proportion of smokers (59.1%), compared to other groups (Table 1).

**Table 1:** Patient Characteristics, demographics and comorbidities.

	No Statin	Low/Moderate intensity	High intensity	*p*‐value
	i = 165	*N* = 1311	*N* = 401	
**Age (Years)**	67.04 ± 10.84	65.23 ± 9.90	64.14 ± 9.49	**0.006** b
**Sex (*n* of Male)**	103 (62.4%)	972 (74.1%)	304 (76.0%)	**0.003** a ^,^ b
**Race/ethnicity (*n*)**				0.616
Caucasian	147 (89.1%)	1,139 (86.9%)	340 (84.8%)	
African American	2 (1.2%)	43 (3.3%)	15 (3.7%)	
Native American	9 (5.5%)	58 (4.4%)	18 (4.5%)	
Hispanic	7 (4.2%)	37 (2.8%)	16 (4.0%)	
Asian	0	20 (1.5%)	5 (1.2%)	
Pacific Islander	0	1 (0.1%)	0	
Multiple	0	6 (0.5%)	4 (1.0%)	
Unknown	0	7 (0.5%)	3 (0.7%)	
**BMI (Kg/m** ^**2**^ **)**	30.95 ± 6.62	30.56 ± 5.81	31.57 ± 5.79	**0.011** c
**Smoker (*n*)**	79 (47.9%)	716 (54.7%)	237 (59.1%)	**0.046** b
**Co‐morbidities (*n*)**				
Hypertension	144 (87.3%)	1,136 (86.7%)	368 (91.8%)	**0.023** c
CVA	18 (10.9%)	102 (7.8%)	29 (7.2%)	0.315
COPD	39 (23.6%)	398 (30.4%)	141 (35.2%)	**0.022** b
Hyperlipidemia	106 (64.2%)	883 (67.4%)	282 (70.3%)	0.327
Diabetes Mellitus	70 (42.4%)	544 (41.5%)	179 (44.6%)	0.536
CKD	27 (16.4%)	220 (16.8%)	63 (15.7%)	0.879
MI	55 (33.3%)	442 (33.7%)	137 (34.2%)	0.978
Stable Angina	110 (66.7%)	834 (63.6%)	253 (63.1%)	0.707
CHF	32 (19.4%)	257 (19.6%)	90 (22.4%)	0.447
PAD	16 (9.7%)	181 (13.8%)	60 (15.0%)	0.248
**HbA1c (%)**	6.47 ± 1.58	6.60 ± 1.55	6.69 ± 1.37	
**GFR**				
> 60 ml/min/1. 73m^2^ (*n*)	111 (67.3%)	934 (71.4%)	293 (73.3%)	0.359
< 60 ml/min/1. 73m^2^ (Avg)	44.46 ± 12.28	44.14 ± 13.36	43.53 ± 14.32	0.894
**Ejection fraction (%)**	54.01 ± 11.34	53.81 ± 11.71	53.24 ± 12.01	0.658
**Previous PCI/Stent (*n*)**	36 (21.8%)	279 (21.3%)	91 (22.7%)	0.833
PCI	31 (18.8%)	256 (19.5%)	79 (19.7%)	0.968
Stent	25 (15.2%)	158 (12.1%)	43 (10.7%)	0.339
**Pre‐CABG medications (*n***)				
ASA	104 (63.0%)	933 (71.2%)	290 (72.5%)	0.064
P2Y12 receptor antagonist	38 (23.0%)	320 (24.4%)	121 (30.3%)	**0.048** c
**Surgical variables**				
Number of grafts (*n*)	3.02 ± 1.09	3.12 ± 0.96	3.05 ± 1.03	0.291
On‐pump surgery (*n*)	119 (73.5%)	963 (75.1%)	282 (71.8%)	0.413
Surgery duration (min)	197.25 ± 66.99	196.67 ± 64.52	188.51 ± 63.46	0.079
**Post ‐CABG medications (*n*)**				
BB	131 (79.4%)	1,144 (87.3%)	358 (89.3%)	**0.006** a ^,^ b
ACE‐I/ARB	79 (47.9%)	737 (56.2%)	235 (58.6%)	0.063
AAD	55 (33.3%)	364 (27.8%)	90 (22.4%)	**0.019** b ^,^ c
CCB	30 (18.2%)	226 (17.2%)	76 (19.0%)	0.723
Statin	0	1,311 (100%)	401 (100%)	**< 0.001** a ^,^ b
ASA	149 (90.3%)	1,257 (95.9%)	383 (95.5%)	**0.006** a ^,^ b
P2Y12 receptor antagonist	86 (52.1%)	804 (61.3%)	244 (60.8%)	0.073
**Survival duration (days)**	916.23 ± 508.15	914.10 ± 1,248.78	879.29 ± 478.91	0.846
**Number of completed rehab sessions (*n*)**	5.04 ± 5.33	5.01 ± 5.19	5.53 ± 6.30	0.275

^a^ Significance between No Statin versus Low/Moderate Intensity.

^b^ Significance between No Statin versus High intensity.

^c^ Significant between Low/Moderate Intensity versus High Intensity.

Hypertension was the most prevalent co‐morbidity (87.3%–91.8%), with a significantly higher proportion in the high‐intensity statin group (91.8%, *p* = 0.023). Chronic obstructive pulmonary disease was more frequent among patients on statin therapy than the no‐statin group (*p* = 0.022). Other co‐morbidities were similar among groups (*p* > 0.05). High intensity statin group was more likely to be prescribed P2Y12 antagonist and beta blockers after CABG than low/moderate intensity and no‐statin groups (Table 1).

## 8 Primary Outcome

**Figure 1:**
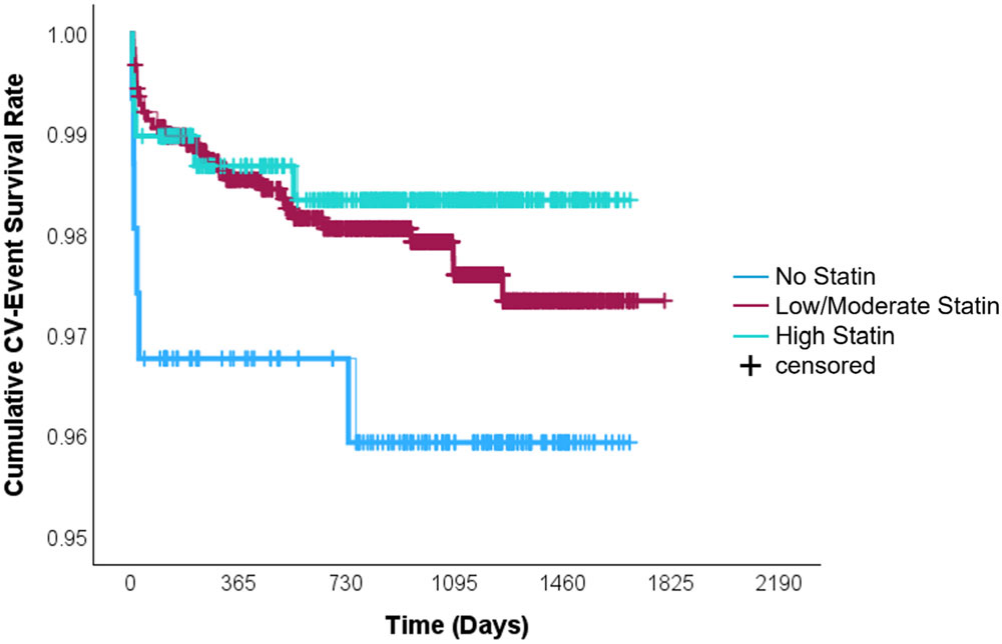
Kaplan‐Meier curves for cardiovascular event‐free survival stratified by statin use and intensity in post‐CABG patients. This figure shows the cumulative cardiovascular (CV) event survival rate over time among patients undergoing coronary artery bypass grafting (CABG), grouped by statin regimen: no statin (light blue), low/moderate‐intensity statin (maroon), and high‐intensity statin (aqua green). High‐intensity statin users exhibited the highest event‐free survival throughout the follow‐up period, while patients not on statins had the poorest outcomes.

The primary outcome of this study was the incidence of MACE following CABG. Patients who died within 7 days were excluded (*n* = 23). The post‐CABG follow‐up period was stratified into two timeframes: early events occurring within 2 years of the index surgery, and late events occurring beyond 2 years. In addition to these time‐based subgroup analyses, overall event rates across the entire follow‐up duration were also measured and analyzed to assess long‐term outcomes across treatment groups. Among 1854 patients, those in no‐statin group (*n* = 156) demonstrated a significantly higher rate of MACE compared to those on statins (n = 1698) over a mean follow‐up period (14.2% vs 8.9%; OR 1.60, 95% CI 1.055–2.427, *p* = 0.029) (Figure 1). Patients not on statin therapy post‐CABG had significantly higher MACE rates beyond 2 years post‐CABG (14.7% vs. 8.2%; OR 1.79, 95% CI: 1.107–2.881; *p* = 0.019) compared to those on statins. (Table 2a).

**Table 2a:** MACE: Statin vs. No Statin.

Timepoint	No statin (*N* = 156)	Statin (N = 1698)	OR	95% CI	*p*‐value
Overall	22 (14.2%)	149 (8.9%)	1.60	1.055–2.427	0.029
After 2 years	17 (14.7%)	98 (8.2%)	1.79	1.107–2.881	0.019

*Note:* Odds ratios (OR) and 95% confidence intervals (CI) compare statin users with non‐users at different timepoints following CABG surgery.

Abbreviation: MACE = Major Adverse Cardiovascular Events.

Primary Outcome – MACE.

Among patients receiving statins, low/moderate‐intensity therapy (*n* = 1301) was associated with a numerically higher overall rate of MACE compared to high‐intensity therapy (*n* = 397), though not statistically significant (9.6% vs 6.6%; OR 1.45, *p* = 0.073). Importantly, beyond 2 years post‐CABG, low/moderate intensity statin use was associated with a significant higher incidence of MACE (9.1% vs 5.3%; OR 1.72, 95% CI 0.993–2.978, *p* = 0.047), compared to higher‐intensity statin therapy (Table 2b).

**Table 2b:** MACE: High‐Intensity vs Low/Moderate‐Intensity Statin.

Timepoint	Low/Moderate intensity (*N* = 1301)	High intensity (*N* = 397)	OR	95% CI	*p*‐value
Overall	123 (9.6%)	26 (6.6%)	1.45	0.961–2.172	0.073
After 2 years	84 (9.1%)	14 (5.3%)	1.72	0.993–2.978	0.047

*Note:* Odds ratios (OR) and 95% confidence intervals (CI) compare high‐intensity vs low/moderate‐intensity statin therapy in post‐CABG patients.

Abbreviation: MACE = Major Adverse Cardiovascular Events.

## 9 Secondary Outcomes

**Figure 2:**
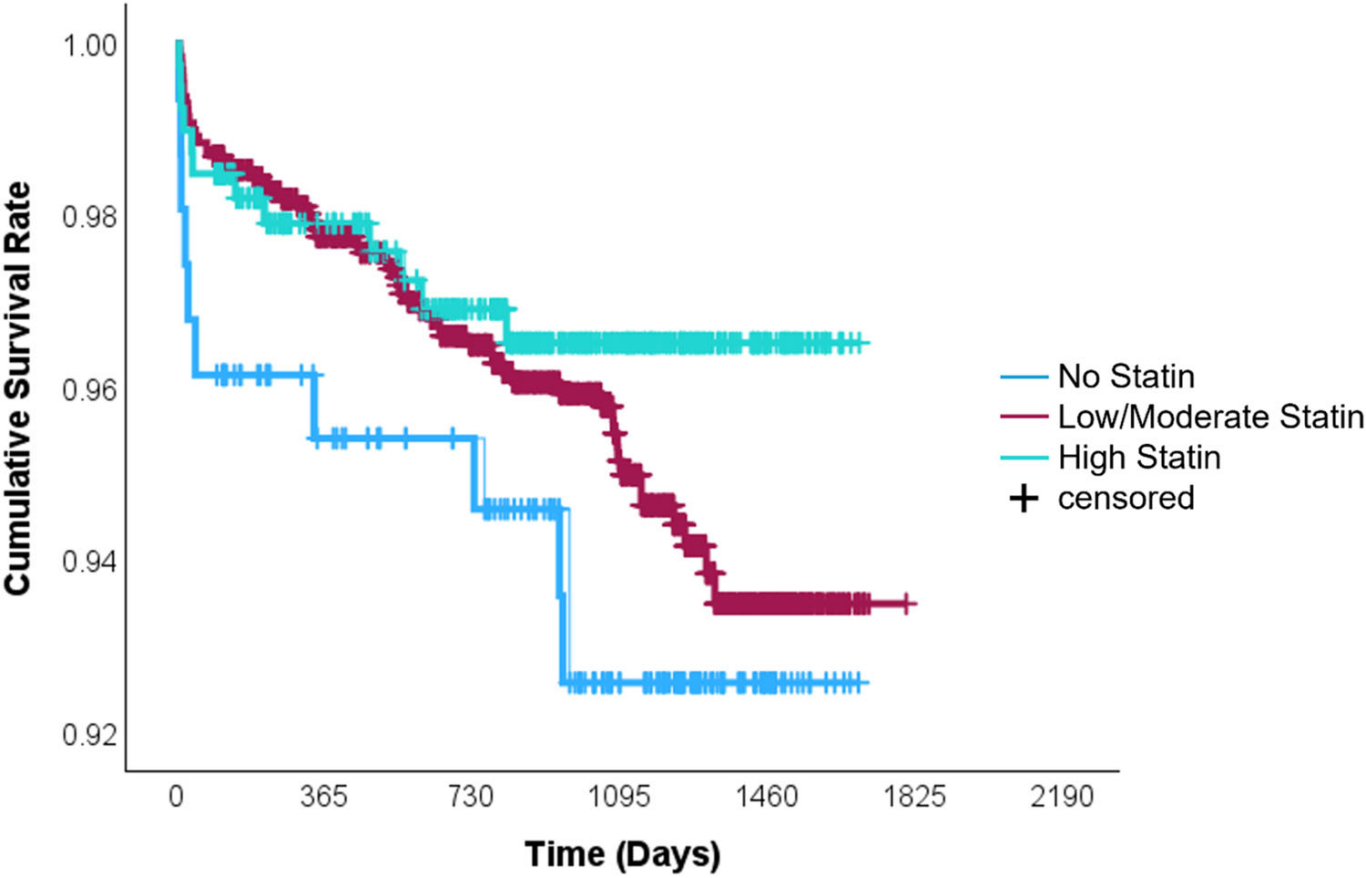
Kaplan‐Meier survival curves showing cumulative survival rates over time (in days) following coronary artery bypass grafting (CABG), stratified by statin use and intensity. Patients were grouped into three categories: no statin (light blue), low/moderate‐intensity statin (maroon), and high‐intensity statin (aqua green). The cumulative survival rate was significantly lower in the no statin group compared to both statin‐treated groups. High‐intensity statin therapy demonstrated the most favorable long‐term survival trend, with the least decline over time. This figure illustrates the survival benefit associated with statin therapy, particularly high‐intensity statins, in post‐CABG patients.

**Figure 3:**
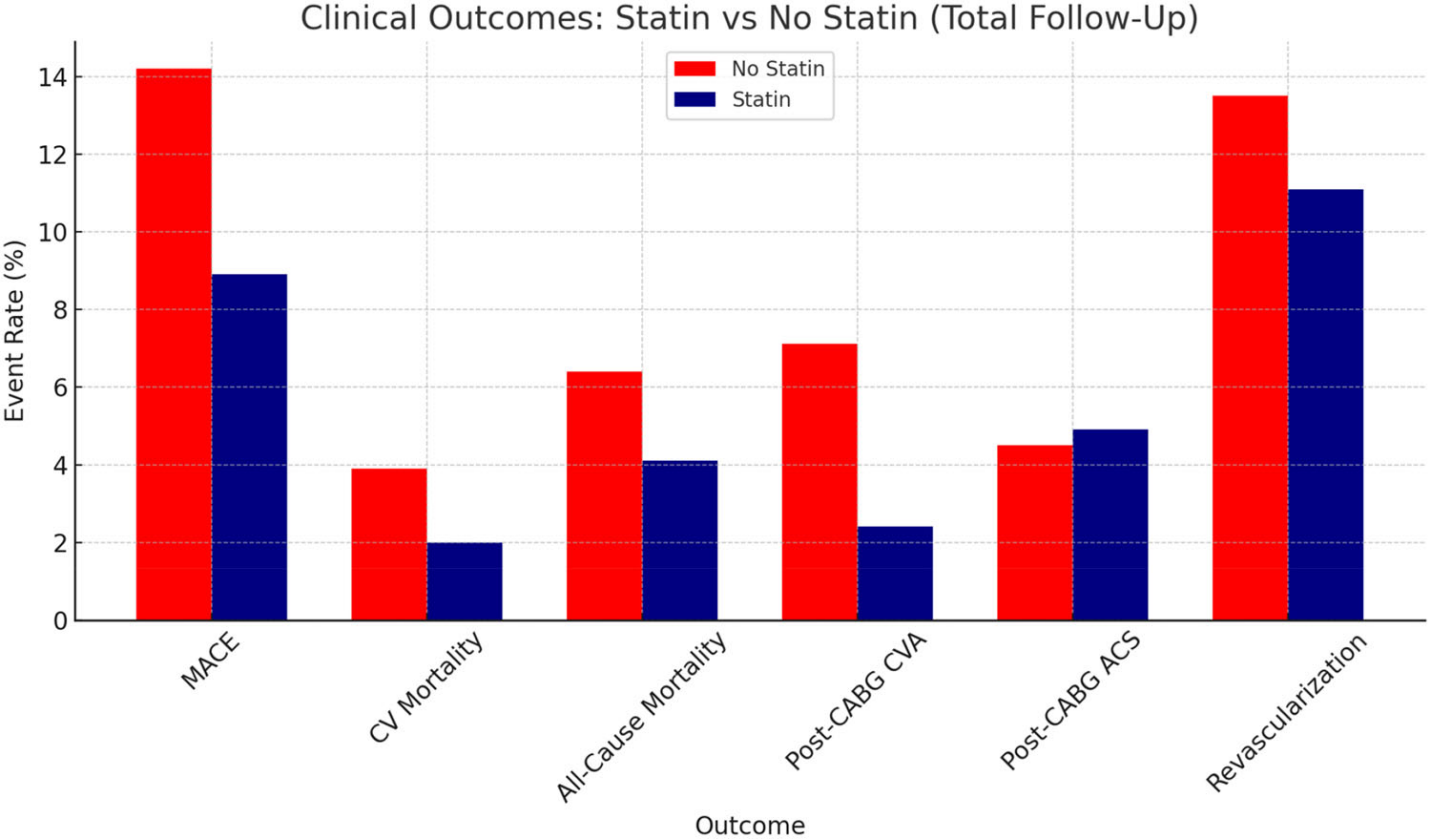
Comparison of clinical outcomes between patients receiving statin therapy versus those not on statins following CABG. Statin use was associated with a reduction in MACE, cerebrovascular accidents (CVA), and mortality. Red bars represent the No Statin group, and Navy Blue bars represent the Statin group. Clinical Outcomes by Statin Use and Intensity.

Secondary outcomes included all‐cause mortality, cardiovascular (CV) mortality, post‐CABG cerebrovascular accident (CVA), acute coronary syndrome (ACS), the need for revascularization and lipid profile (Table 3). While statin therapy was associated with numerically lower rates of all‐cause mortality (4.1% vs 6.4%, *p* = 0.178) and CV mortality (2.0% vs 3.9%, *p* = 0.116) (Figure 2) compared to the no‐statin group, these differences did not reach statistical significance (Figures 1 and 2). The incidence of post‐CABG CVA was higher among no‐statin group versus statin users (7.1% vs 2.4%, OR 2.99, 95% CI 1.568–5.716, *p* < 0.001). Temporal analysis revealed beyond 2 years, no‐statin group continued to have higher incidence of post‐CABG CVA (8.6% vs 2.7%, OR 3.24, 95% CI 1.633–6.412, *p* < 0.001). While mortality, rates of ACS and revascularization were similar between groups (Figure 3) (Table 4a).

**Table 3:** Impact of Statin Intensity on Lipid Profile Post‐CABG.

Post‐CABG Lipid Panel (mg/dL)	Low Intensity statin	Moderate intensity statin	High Intensity statin	
LDL	104.83 ± 38.93	90.84 ± 45.89	82.21 ± 41.85	< 0.001^a^^,^ b ^,^ c
HDL	42.05 ± 12.34	42.42 ± 18.29	41.14 ± 12.94	0.477
Total cholesterol	179.25 ± 65.21	163.92 ± 84.30	155.86 ± 57.53	0.017^b^
Triglycerides	170.11 ± 72.76	166.73 ± 110.28	181.71 ± 222.57	0.234

Legend: LDL = Low‐Density Lipoprotein; HDL = High‐Density Lipoprotein. Lipid values reflect postoperative profile by statin intensity.

^a^ Significance between No Statin versus Low/Moderate Intensity.

^b^ Significance between No Statin versus High intensity.

^c^ Significant between Low/Moderate Intensity versus High Intensity.

**Table 4a:** Secondary Outcomes by Statin Use (Overall, ≤ 2 Years, > 2 Years).

Outcome	Timing	No statin (N = 156)	Statin (N = 1,698)	OR	95% CI	*p*‐value
CV mortality	Overall	6 (3.9%)	33 (2.0%)	1.97	0.837–4.622	0.116
All‐cause mortality	Overall	10 (6.4%)	70 (4.1%)	1.59	0.804–3.157	0.178
Post‐CABG CVA	Overall	11 (7.1%)	40 (2.4%)	2.99	1.568–5.716	< 0.001
ACS	Overall	7 (4.5%)	84 (4.9%)	0.91	0.427–1.927	0.799
Revascularization	Overall	21 (13.5%)	188 (11.1%)	1.22	0.799–1.851	0.366
CV mortality	> 2 years	1 (0.9%)	4 (0.3%)	2.57	0.290–22.832	0.379
All‐cause mortality	> 2 years	3 (2.26%)	18 (1.5%)	1.75	0.506–6.014	0.372
Post‐CABG CVA	> 2 years	10 (8.6%)	32 (2.7%)	3.24	1.633–6.412	< 0.001
ACS	> 2 years	7 (6.0%)	65 (5.4%)	1.12	0.524–2.375	0.778
Revascularization	> 2 years	19 (16.4%)	163 (13.6%)	1.21	0.781–1.865	0.403

*Note:* Secondary Outcomes Post‐CABG: Statin Use.

Abbreviations: ACS = Acute Coronary Syndrome, CI = Confidence Interval, CV = Cardiovascular, CVA = Cerebrovascular Accident, OR = Odds Ratio.

**Figure 4:**
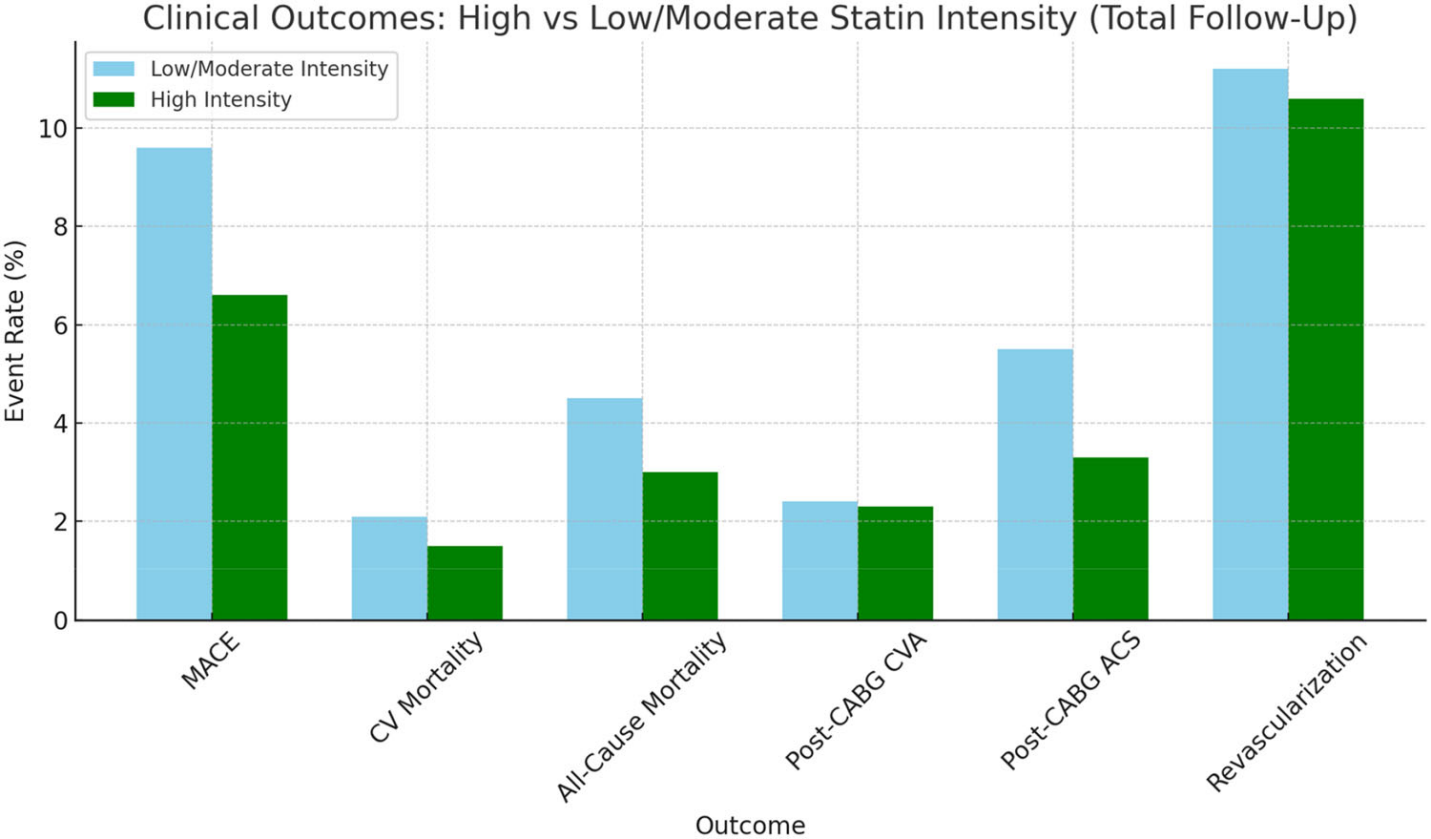
Comparison of clinical outcomes between patients receiving high‐intensity statins versus low/moderate‐intensity statins. Green bars represent the High Intensity group, and Sky Blue bars represent the Low/Moderate Intensity group. Outcomes include MACE, CV and all‐cause mortality, post‐CABG ACS, CVA, and revascularization.

Among patients on statins, low/moderate intensity therapy was associated with numerically higher, though nonsignificant, rates of all‐cause mortality (4.5% vs 3.0%, OR 1.50, *p* = 0.208), CV mortality (2.1% vs 1.5%, OR 1.38, *p* = 0.472), and post‐CABG CVA (2.4% vs 2.3%, OR 1.05, *p* = 0.894) compared to low/moderate‐intensity therapy (Figure 4) (Table 4b). No significant differences were found in ACS or revascularization rates between intensity groups across the follow‐up duration.

**Table 4b:** Secondary Outcomes by Statin Intensity (Overall, ≤ 2 Years, > 2 Years).

Outcome	Timing	Low/Mod Intensity (*N* = 1301)	High Intensity (*N* = 397)	OR	95% CI	*p*‐value
CV mortality	Overall	27 (2.1%)	6 (1.5%)	1.38	0.573–3.312	0.472
All‐cause mortality	Overall	58 (4.5%)	12 (3.0%)	1.50	0.796–2.816	0.208
Post‐CABG CVA	Overall	31 (2.4%)	9 (2.3%)	1.05	0.505–2.188	0.894
ACS	Overall	71 (5.5%)	13 (3.3%)	1.67	0.933–2.978	0.079
Revascularization	Overall	146 (11.2%)	42 (10.6%)	1.06	0.767–1.467	0.721
CV Mortality	> 2 years	4 (0.4%)	0	NS	—	0.283
All‐cause mortality	> 2 years	17 (1.8%)	1 (0.4%)	4.93	0.653–37.226	0.086
Post‐CABG CVA	> 2 years	26 (2.8%)	6 (2.2%)	1.24	0.515–2.978	0.631
ACS	> 2 years	56 (6.0%)	9 (3.4%)	1.78	0.892–3.548	0.095
Revascularization	> 2 years	128 (13.7%)	35 (13.1%)	1.05	0.738–1.481	0.802

Abbreviations: ACS = Acute Coronary Syndrome, CI = Confidence Interval, CV = Cardiovascular, CVA = Cerebrovascular Accident, OR = Odds Ratio.

Secondary Outcomes Post‐CABG: Statin Intensity.

## 10 Impact of Different Statin Therapy on Lipid Management

Patients who received high‐intensity statin therapy had the lowest LDL levels (82.21 ± 41.85 mg/dL), followed by those on low/moderate‐intensity statins (90.84 ± 45.89 mg/dL), while the no‐statin group exhibited the highest LDL levels (104.83 ± 38.93 mg/dL, *p* < 0.001). Similarly, total cholesterol was significantly lower in the high‐intensity statin group (155.86 ± 57.53 mg/dL) compared to low/moderate‐intensity (163.92 ± 84.30 mg/dL) and no‐statin (179.25 ± 65.21 mg/dL) groups (*p* = 0.017), indicating better lipid control with more intensive statin therapy (Table 4).

In contrast, HDL levels were similar across all groups, with no statistically significant difference (*p* = 0.477). Likewise, triglyceride levels did not differ significantly among the groups (*p* = 0.234), suggesting that statin therapy had a more pronounced effect on LDL and total cholesterol reduction rather than HDL elevation or triglyceride lowering.

## 11 Discussion

There is extensive evidence supporting the use of high‐intensity statin therapy for secondary prevention in patients with clinical atherosclerotic cardiovascular disease [3, 4, 5, 8]. However, whether initiating high‐intensity statins early after CABG provides additional benefits in clinical outcomes, graft patency, or slowing vein graft disease progression compared to low to moderate‐intensity statins remains uncertain, emphasizing the need for further investigation. In this observational study, we examined the impact of statin intensity on post‐CABG clinical outcomes at a high‐volume, single‐center open‐heart surgery program.

## 12 Patient Characteristics

The majority of patients (90.6%) at our center received statin therapy following CABG surgery, with approximately 21% prescribed high‐intensity statins. Patients who were not on statins, often due to intolerance or side effects, were on average 2 years older (mean age: 67.04 years) than those in the statin‐treated groups. Additionally, statin intensity was inversely related to age (*p* = 0.0062), suggesting that younger patients may have better tolerance to high‐intensity statins. A significant gender disparity was evident, with women being less likely to receive statins and more frequently prescribed lower‐intensity therapy compared to men (Table 1). This finding raises concerns about potential sex‐based differences in cardiovascular care, which may stem from variations in medication tolerance, adherence, or prescribing practices.

Patients on high‐intensity statins had distinct baseline characteristics that likely influenced both treatment decisions and clinical outcomes. This group had a higher BMI (31.57 ± 5.79 kg/m²), a greater proportion of smokers (59.1%), and a higher prevalence of hypertension and chronic obstructive pulmonary disease —suggesting a greater overall burden of comorbidities. Consequently, these patients may have been targeted for more aggressive secondary prevention strategies. Supporting this notion, the use of P2Y12 antagonists and beta‐blockers was more common in the high‐intensity statin group, reflecting a comprehensive approach to post‐CABG care in this cohort.

## 13 Primary Outcome

Our findings reinforce the critical role of statin therapy in reducing MACE following CABG. Patients receiving statins experienced significantly lower MACE rates compared to those not on statins, highlighting the importance of lipid‐lowering therapy in secondary prevention. Notably, this benefit was more evident beyond the first 2 years of post‐surgery, suggesting that the long‐term protective effects of statins may take time to fully manifest. While high‐intensity statin therapy showed a numerical advantage over low/moderate‐intensity regimens in overall MACE reduction, statistical significance was reached only in the late post‐CABG period. This supports the notion that the benefits of high‐intensity statins may be cumulative and more pronounced with extended follow‐up. These results underscore the importance of both initiating and maintaining statin therapy—particularly at higher intensities where tolerated—to improve long‐term cardiovascular outcomes in CABG patients.

Several factors may have impacted outcomes: First, patient Selection and Tolerance: high‐intensity statins were more commonly prescribed to patients with higher BMI, smoking history, and more comorbidities (hypertension, lung disease)—a population with potentially greater cardiovascular risk. The difference may reflect underlying risk factors mitigating the benefit of higher‐intensity therapy and in a matched study may yield better outcomes. Second, while high‐intensity statins are recommended for secondary prevention, tolerance and adherence issues may limit their effectiveness, leading to dose reductions or discontinuation over time. We didn't collect data on compliance with these medications over time, which may have introduced bias in this study. Third, the benefits of statin intensity appear to be more long‐term, as indicated by findings from this study and aligns well with known literature; several RCTs and meta‐analyses [4, 5, 8, 9] reported superiority of high intensity therapy in patients with chronic coronary disease—including CABG patients. It may be suggested that high intensity statin clinical benefits are long term and should be the first choice of treatment consistent with current guidelines. While certain clinical or postoperative limitations may restrict the use of high‐intensity statins in the immediate post‐CABG period, every effort should be made to initiate high‐intensity statin therapy as early as feasible to optimize long‐term cardiovascular outcomes. This observational study, conducted on a relatively large sample, provides robust evidence that should encourage clinicians to consider utilizing higher‐intensity medication when feasible for treating high‐risk patients, such as those who have undergone CABG surgery.

## 14 Secondary Outcomes

In this study, statin therapy following CABG was associated with favorable trends across several secondary endpoints. Although the observed reductions in all‐cause mortality (4.1% vs 6.4%; *p* = 0.178) and cardiovascular mortality (2.0% vs 3.9%;, *p* = 0.116) in the statin‐treated group did not reach statistical significance, the directionality of these effects aligns with existing evidence supporting the prognostic benefits of statins in secondary prevention.

A notable and statistically significant reduction in post‐CABG CVA was observed among patients receiving statins (2.4% vs 7.1%, *p* < 0.001), highlighting the cerebroprotective role of statin therapy. Temporal analysis demonstrated that this benefit was sustained even at long term follow up. After 2 years post‐CABG, statin therapy remained significantly associated with lower incidence of CVA (2.7% vs 8.6%, *p* < 0.001), whereas differences in all‐cause and cardiovascular mortality remained nonsignificant.

Among patients receiving statins, comparison of high‐intensity versus low/moderate‐intensity therapy revealed no statistically significant differences in secondary outcomes, including mortality, CVA, ACS, or need for revascularization. Nevertheless, numerically lower event rates in the high‐intensity group suggest a potential long‐term benefit that may be underpowered in this analysis but warrants further investigation. These findings support the broader cerebrovascular benefits of statin therapy and reinforce the importance of long‐term risk modification in post‐CABG patients. The observed reduction in MACE among statin users compared to non‐users, along with the greater long‐term MACE reduction in the high‐intensity statin group versus low/moderate‐intensity, provides clear direction for surgeons and clinicians to prioritize statin intensity. These findings highlight not only early potential benefits but also pronounced long‐term advantages, supporting a strategy of high‐intensity statin use following CABG to improve clinical outcomes.

## 15 Impact of Different Statin Therapy on Lipid Management

Our findings underscore the efficacy of statin therapy in achieving superior lipid control, particularly in reducing LDL and total cholesterol levels. Patients receiving high‐intensity statins exhibited the lowest LDL levels (82.21 ± 41.85 mg/dL), followed by those on low/moderate‐intensity statins (90.84 ± 45.89 mg/dL), while the no‐statin group had the highest LDL levels (104.83 ± 38.93 mg/dL, *p* < 0.001). Similarly, total cholesterol was significantly lower in the high‐intensity statin group (155.86 ± 57.53 mg/dL) compared to low/moderate‐intensity (163.92 ± 84.30 mg/dL) and no‐statin (179.25 ± 65.21 mg/dL) groups (*p* = 0.017). LDL reduction was significantly better with both high‐intensity and low/moderate‐intensity statins compared to no statins. However, total cholesterol reduction was only significant with high‐intensity statins (Table 3). These results reinforce the dose‐dependent lipid‐lowering effects of statins, with more intensive therapy leading to greater reductions in atherogenic lipid parameters. The absence of significant differences in other parameters is consistent with prior literature, which indicates that HDL modulation and triglyceride reduction are less pronounced effects of statin therapy, and other lipid‐modifying agents (such as fibrates or PCSK9 inhibitors) may be required in patients with persistent dyslipidemia beyond LDL reduction. Statin therapy exerts beneficial effects beyond lipid lowering [10], pleiotropic effects of statins including plaque stabilization, improved endothelial function, anti‐inflammatory and anti‐thrombotic activity as well as immune mediated mechanisms ‐ all of which contribute to lowering MACE and improved clinical outcomes [11, 12, 13].

## 16 Comments

Several RCTs and meta‐analyses have demonstrated the long‐term benefits of high‐intensity statin therapy in patients with prior CABG. However, a critical limitation of many of these studies is the delayed timing of patient recruitment—often months to years after index surgery. As a result, these investigations fail to capture the early postoperative period, during which unique physiological factors such as graft healing, surgical stress, and systemic inflammation may significantly impact cardiovascular outcomes. Our study aimed to explore this specific question. Although prior trials such as the Post‐CABG trial, PROVE IT‐TIMI 22, and A to Z [7, 8, 9] reported favorable outcomes with intensive statin therapy, these studies had notable limitations. In many cases, the inclusion of post‐CABG patients was not predefined, and the number of CABG patients enrolled was relatively small. Additionally, the intensity of statin therapy used in these earlier trials often did not align with current standards, as these studies were conducted decades ago. These gaps underscore the need for more contemporary and focused research to better understand the role of statin intensity in the immediate postoperative period following CABG.

Cholesterol Treatment Trialists' (CTT) meta‐analysis reported a 15% reduction in MACE with high‐intensity statins, particularly among older patients, and highlighted greater benefit with greater LDL reduction [4]. Likewise, aggressive long‐term lipid‐lowering strategies have shown efficacy in slowing atherosclerosis progression in saphenous vein grafts and reducing myocardial infarction and revascularization rates. Nonetheless, these findings largely reflect outcomes in CCD populations and may not be generalizable to the immediate post‐CABG context. Notably, a post hoc analysis from a randomized trial that included CABG patients reported favorable effects of high‐intensity statins, but interpretation was limited by the lack of a pre‐specified CABG subgroup, absence of key prognostic variables (e.g., graft type and extent of coronary disease), and delayed enrollment well beyond the operative period [14]. Similar methodological constraints have been observed in other studies and meta‐analyses evaluating statin intensity in post‐CABG patients [15, 16, 17]. These limitations highlight the need for targeted research that evaluates statin intensity beginning in the early postoperative phase.

Our observational study aimed to address a key evidence gap by evaluating the role of high‐intensity statin therapy initiated soon after CABG. The findings favor the use of high‐intensity statins where feasible, supporting their broader implementation in the early postoperative setting. In addition to achieving superior lipid lowering, as consistently reported across multiple studies, high‐intensity statins may offer additional clinical benefits—including potential reductions in post‐CABG atrial fibrillation and improved graft patency outcomes through their pleiotropic effects [17, 18, 19].

High‐intensity statin therapy is clearly associated with more robust LDL reduction compared to low‐ or moderate‐intensity regimens. Elevated LDL levels within the first postoperative year have been linked to an increased risk of subsequent vein graft disease years after CABG [6, 20]. Intracoronary angioscopy studies further corroborate the significance of LDL lowering, showing the presence of plaque and thrombus in grafts of patients with LDL > 100 mg/dL, whereas those with LDL < 80 mg/dL had no such findings [21]. Similarly, post hoc analysis of the CASCADE trial revealed significantly higher 1‐year graft patency rates in patients with LDL < 100 mg/dL compared to those with higher levels (96.5% vs 83.3%, *p* = 0.03), although further reduction to < 70 mg/dL provided no added benefit [15]. Of note, the COSMOS trial demonstrated improved graft outcomes with high‐intensity statins independent of LDL levels, suggesting additional mechanisms of benefit [22].

## 17 Limitations

This study has several important limitations. First, it is a single‐center observational study, which may limit the generalizability of the findings and introduce potential institutional biases. Second, due to its non‐randomized design, we were unable to perform matched comparisons between groups, and residual confounding cannot be excluded despite statistical adjustments. Third, we did not have longitudinal data to confirm long‐term adherence and compliance with statin therapy, which may have influenced clinical outcomes and attenuated the observed effects. Additionally, lipid profiles were recorded between one to 3 months after surgery for most patients, and this variability in timing may have introduced measurement bias, potentially impacting one group more than the other. These limitations highlight the need for prospective, multicenter studies to validate our findings and to better evaluate the role of statin intensity during the early and late postoperative phases following CABG.

## 18 Conclusion

In this large single‐center cohort of post‐CABG patients, statin therapy was associated with a significant reduction in major adverse cardiovasocular events, particularly beyond the early postoperative period. High‐intensity statins showed additional long‐term benefit, supporting their preferred use in patients after CABG.

## Ethics Statement

This ethics statement affirms that the research adhered to the highest ethical standards and guidelines established by regulatory bodies. Informed consent was obtained from the participants, confidentiality and privacy of participant information were maintained, conflicts of interest were denied, high morals were upheld, data integrity and transparency were ensured, authorship criteria were outlined, and contributions were acknowledged appropriately.

## Conflicts of Interest

There are no conflicts related to this manuscript. Dr. Khurram Nasir is on advisory board of Amgen, Regeneron, Amsterdam Pharma, Merck Sharp & Dolme. Research is partly supported by grants from NIH, PCORI, Novartis and Ionis.

## Data Availability

The data supporting the findings of this study are available upon reasonable request. However, restrictions apply as these data were used under license for this study and cannot be made publicly accessible. The hospital's policy strictly prohibits sharing patient data online to avoid potential breaches of confidentiality and violations of applicable data protection laws. In accordance with these regulations, the full data set cannot be publicly shared. Nevertheless, researchers may request access to the data by contacting the authors and obtaining permission from Oklahoma Heart Hospital.
